# Association between serum lipid dysregulation, management, and cognitive decline in rural and urban Southern Indian population

**DOI:** 10.1002/alz70860_107279

**Published:** 2025-12-23

**Authors:** Priya Chatterjee, Jonas S Sundarakumar, Thomas Gregor Issac, CBR‐SANSCOG and CBR‐TLSA study teams

**Affiliations:** ^1^ Centre for Brain Research, Indian Institute of Science, Bangalore, Karnataka, India

## Abstract

**Background:**

Previous studies have shown that abnormal lipid levels in midlife are associated with poorer cognitive performance and increased risk of dementia in later life. However, this relationship remains underexplored in the Indian population.

**Method:**

We analyzed longitudinal data from two ongoing cohort studies: Centre for Brain Research‐Srinivaspura Aging NeuroSenescence and COGnition (CBR‐SANSCOG, *n* = 766) and CBR‐Tata Longitudinal Study of Aging (CBR‐TLSA, *n* = 518) comprising individuals aged ≥45 years without dementia from a rural and an urban population from southern India, respectively. Baseline serum lipid profiles were measured from fasting venous blood samples. Cognitive assessments were conducted at baseline and follow‐up using the culturally adapted Computerized assessment of adult information processing (COGNITO) test battery. Individual test scores were standardized. Multiple linear regression models, adjusting for appropriate covariates, were used to study the association between the baseline lipid parameters and the rate of change in cognitive performance. The rate of change in cognitive performance was calculated by subtracting the first follow‐up cognitive test scores from the baseline scores and dividing this difference by the time interval between the two assessments (mean duration of follow‐up: rural=2.6 years, urban = 1 year). The use of cholesterol‐lowering medication was considered for the urban population but not the rural population as less than 1% of people reported using these drugs.

**Result:**

Higher total cholesterol[mg/dl] level was associated with a steeper decline in the memory domain[delayed recall, β=0.002, 95%CI (0.001, 0.003)] in the rural population. No significant association was found between the lipid parameters and cognitive performance in the urban population. However, in this population, the use of cholesterol‐lowering medication was associated with a slower decline in language[‐0.378,(‐0.649,‐0.106)] and memory (immediate [‐0.369,(‐0.642,‐0.097)] and delayed [‐0.439, (‐0.711, ‐0.166)] recall) functioning and a steeper decline of visual attention[0.393,(0.120,0.665)].

**Conclusion:**

In the rural population, we observed a steeper cognitive decline in individuals with higher total cholesterol. Additionally, we report a complex effect of cholesterol‐lowering medication on cognitive performance in the urban cohort. Further research on the effects of lipid abnormalities and the impact of dose, duration, and class‐specific effects of lipid‐lowering treatments on cognitive trajectory needs to be prioritized in diverse populations for better cognitive outcome in these populations.

